# Cryogenic, but not hypothermic, preservation disrupts the extracellular matrix of cell sheets

**DOI:** 10.1016/j.bioactmat.2024.12.019

**Published:** 2024-12-25

**Authors:** Sara Freitas-Ribeiro, Andreia F. Carvalho, Daniel B. Rodrigues, Luís Martins, Ricardo A. Pires, Vera M. Mendes, Bruno Manadas, Mariana Jarnalo, Ricardo Horta, Rui L. Reis, Rogério P. Pirraco

**Affiliations:** a 3B's Research Group, I3Bs – Research Institute on Biomaterials, Biodegradables and Biomimetics, University of Minho, Headquarters of the European Institute of Excellence on Tissue Engineering and Regenerative Medicine, AvePark, Parque de Ciência e Tecnologia, Rua Ave 1, Edifício 1 (Sede), 4805-694 Barco, Guimarães, Portugal; b ICVS/3B's–PT Government Associate Laboratory, Braga/Guimarães, Portugal; c Instituto de Ciências Biomédicas Abel Salazar (ICBAS), Universidade Do Porto, Porto, Portugal; d CNC-Center for Neuroscience and Cell Biology, University of Coimbra, Coimbra, Portugal; e Institute for Interdisciplinary Research, University of Coimbra, Coimbra, Portugal; f Department of Plastic and Reconstructive Surgery, and Burn Unity, Centro Hospitalar de São João, Porto, Portugal; g Faculty of Medicine - University of Porto, Portugal

**Keywords:** Cell sheets, Extracellular matrix, Biopreservation, Hypothermic preservation, Cryogenic preservation

## Abstract

Cell sheet (CS)-based approaches hold significant potential for tissue regeneration, relying on the extracellular matrix (ECM) for success. Like in native tissues, the ECM provides structural and biochemical support for cellular homeostasis and function. Effective preservation strategies that maintain ECM integrity are critical to enhance the therapeutic potential of CS-based approaches. While cryogenic and hypothermic preservation methods offer potential solutions, their impact on CS ECM structure is not fully understood. Therefore, a comprehensive analysis of the ECM of hASCs CS following cryogenic and hypothermic preservation for 3 and 7 days, was conducted. Although proteomic analysis indicated that cryopreservation had no significant effect on the overall composition of the ECM, it induced significant ECM structural alterations, particularly disrupting collagen organization, which was not observed following hypothermic preservation. These structural changes were accompanied by alterations in mechanical properties, including a reduction in elastic modulus. In contrast, hypothermic preservation maintained ECM integrity and mechanical properties similar to the control. The notable ECM structural changes following cryogenic preservation can potentially impact cellular behavior, including adhesion, proliferation, and differentiation, thereby affecting the efficacy of CS therapies in vivo. This suggests that hypothermia may offer a promising alternative to cryopreservation for preserving CS integrity and functionality.

## Highlights

• Cold preservation strategies did not influence ECM protein abundance.
• Cryogenic temperatures resulted in alterations in ECM structure and mechanical properties.
• Hypothermic preservation succesfully maintained extracellular matrix integrity and mechanical properties.

## Graphical abstract

**Figure:**
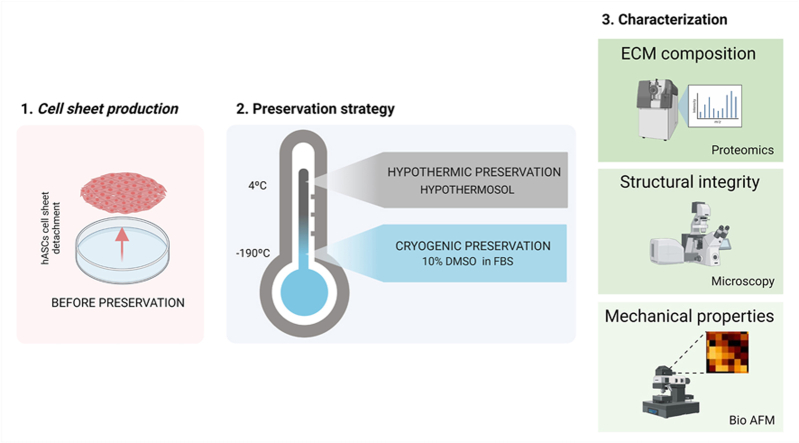

## 1 Introduction

The extracellular matrix (ECM) is a vital component of every tissue. It consists of a large variety of macromolecules whose composition and specific structures vary from tissue to tissue. The main constituents of the ECM are proteoglycans and fibrous proteins, of which collagens, elastins, fibronectins and laminins are the most abundant [1]. These components associate to form the complex network that constitutes the physico-chemical microenvironment where cells live, providing them structural support as well as environmental signals, which may lead to proliferation, differentiation, or death [1].

Considering its importance to so many fundamental cellular processes, the ECM is a crucial part of every tissue engineering and regenerative medicine strategy. Cell Sheet (CS) Engineering-based strategies, in particular, rely heavily on cell-secreted ECM, which is the main constituent of CS constructs. From single-cell sheets to multilayered constructs, using one or more cell types, numerous studies have shown the potential of these ECM-rich constructs for the regeneration of a wide range of tissues [2] such as cornea [3], myocardium [4], articular cartilage [5], bone [6] and skin [7]. However, the widespread clinical application of these constructs will depend on developing successful methodologies that preserve CS properties, particularly the ECM, from the fabrication site to the bedside. Cryogenic preservation and hypothermic preservation emerge as potential candidates for that purpose.

Slow freezing is the most traditional form of cryogenic preservation and has mainly been used to preserve single-cell suspensions [[8], [9], [10], [11]]. Cryoprotectant diffusion and uniform cooling occur easily in cell suspensions, protecting cells from ice formation. Due to their thickness, this is more difficult to achieve in complex systems such as tissues and organs. ECM damage following cryopreservation has been observed in several tissues, mainly in collagen and elastin of heart valves [12] and loss of layered structure [13] in corneal epithelial CSs.

Hypothermic temperatures have long been used in several medical applications [14,15], namely organ preservation during transportation from donor to recipient [16]. These temperatures slow down energy-dependent processes like protein synthesis, transport systems, and cell cycle progression. This way, cells can be suspended during a short period of time with a simplified method, avoiding cellular and structural damage from ice formation and changes in solute concentration caused by extreme temperature shifts during freezing. However, prolonged exposure to hypothermic temperatures can have some damaging effects, especially at the cellular level [17,18].

In this work, a direct comparison of the effects of cryogenic vs hypothermic preservation on the ECM of CSs was performed by looking at putative changes on ECM composition and structure following both preservation methodologies. For each preservation method, the gold standard preservation solution was used. Human adipose stromal cells (hASCs) CSs were either stored at −196 °C using fetal bovine serum (FBS) with 10 % (v/v) dimethyl sulfoxide (DMSO), or at 4 °C using the preservative hypothermosol (HTS). After 3 and 7 days of preservation, ECM protein abundance, structural integrity and mechanical properties were assessed.

## 2 Materials and methods

### 2.1 Isolation of human adipose stromal cells

Human subcutaneous adipose tissue was obtained from surgical procedures performed at Centro Hospitalar Universitário São João, after patient's written informed consent, and in the scope of a collaboration protocol approved by the ethical committees of both institutions for this work (Comissão de Ética do Centro Hospitalar Universitário São João/University of Minho: 217/19; CEICVS 008/2019). hASCs were obtained as previously described [19]. Briefly, after being cut into small pieces, adipose tissue was digested with a solution of 0.05 % (w/v) collagenase type II (Sigma Aldrich, USA) for 45 min at 37 °C under agitation and then centrifuged to obtain stromal vascular fraction (SVF). The obtained SVF was incubated with red blood lysis buffer, centrifuged and the supernatant resuspended in Minimum Essential Medium alpha-modification (α-MEM) (Life Technologies, United Kingdom) supplemented with 10 % (v/v) FBS and 1 % (v/v) antibiotic/antimycotic. The nucleated cells were counted using a solution of 3 % (v/v) acetic acid (VWR, United Kingdom) and 0.05 % (v/v) methylene blue (Sigma Aldrich, USA) in water, plated in culture flasks (Falcon, United Kingdom) in the same medium as before, expanded in culture with frequent medium changes and passaged after reaching 80 % confluence. Cells were used up to passage 4.

### 2.2 Cell sheets production and detachment

At the beginning of each experimental setup, cells were detached, centrifuged and plated in 6 well tissue culture polystyrene (TCPS) plates (Falcon, United Kingdom) at a density of 31,500 cells/cm^2^. hASCs were then grown for 5 days with α-MEM supplemented with 50 μg/mL ascorbic acid (Wako, USA) to produce CS. CS were then detached by mechanical manipulation from the plates. Briefly, the medium was replaced by Dulbecco's phosphate buffer saline (DPBS) (Thermo Scientific, USA), and forceps were used for mechanical peeling. After, CS were either subjected to the different preservation studies (hypothermic and cryogenic) or characterized immediately, forming in this case the "before preservation" (BP) group.

### 2.3 Hypothermic preservation

After CS detachment, DPBS was replaced with hypothermosol (BioLife Solutions, USA) and CS were placed at 4 °C. After a storage period of 3 and 7 days, CS were washed with DPBS and characterized immediately, forming the HP group.

### 2.4 Cryogenic preservation

Following detachment, DPBS was removed, and a poly(vinylidene difluoride) (PVDF) membrane (Millipore Corporation, U.S.A.) was placed over the CS to allow their manipulation. CSs were then placed in a Teflon container with a solution of FBS with 10 % (v/v) DMSO, frozen at −20 °C for 2h and −80 °C overnight. Finally, the containers were stored at −190 °C in a dry vapour phase cryotank (Biosystem Arctic 24 statebourne cryogenics, USA). After 3 and 7 days of storage, CSs were washed with DPBS and characterized immediately, forming the CP group.

### 2.5 ECM extraction

CSs were decellularized to assess ECM composition. CSs were transferred to a microcentrifuge tube and incubated with a solution of 20 mM NH_4_OH and 1 % (v/v) Triton x-100 in water (all from Sigma Aldrich, USA) for 3 min at 37 °C. After a centrifugation step, the remaining pellet was incubated with 200U DNase (Thermo Scientific, USA) and cOmplete protease and phosphatase inhibitors mini tablets (Merck, Germany) according to manufacturer instructions. Samples were centrifuged, the supernatant discarded, and the pellet solubilized in SDS buffer (7 % (w/v) 0.5 M Tris.HCl/0.4 % SDS pH 6.8 buffer), 30 % (w/v) glycerol, 10 % (w/v) SDS, 0,93 % (w/v) DTT and 0.012 % (w/v) bromophenol blue, in water). Finally, samples were incubated for 20 min at 40 °C, 20 min at 65 °C and 10 min at 90 °C. Total protein was quantified using a micro Pierce 660 nm Protein Assay Reagent with the Ionic Detergent Compatibility Reagent (all from Thermo Scientific Pierce, USA), according to the manufacturer instructions.

### 2.6 Proteomics

Short GeLC-SWATH-MS (Sequential Window Acquisition of all Theoretical Mass Spectra/Data Independent Acquisition - DIA) was performed as previously described [20]. Briefly, 30 μg of each sample was subjected to in-gel digestion after a partial SDS-PAGE run using precast gel (4–20 % Mini-Protean® TGX™ Gel, Bio-Rad). Mass spectrometry data were acquired in two different acquisition modes: Data-Dependent Acquisition (DDA) of the pooled samples and DIA of each individual sample. Protein identification and library construction were performed using ProteinPilot™ (v5.0.1, Sciex), and the relative quantification was performed using SWATH™ processing plug-in for PeakView™ (v2.2, Sciex).

### 2.7 Histological analysis

After formalin fixation with 10 % (v/v) buffered formalin solution, CSs were embedded in histogel (Thermo Scientific, USA) to protect them during the paraffin embedding process. CSs were then processed in a MICRON STP120-2 spin tissue processor (MICRON, Germany), embedded in paraffin (Thermo Scientific, USA), and serially sectioned into 4 μm-thick sections.

### 2.8 Immunohistochemistry

CS sections were deparaffinized and rehydrated in a MICROM HMS740 automatic stainer (MICROM, Germany). Antigen retrieval was performed by treating the sections with citrate buffer at 95 °C for 4 min, followed by alizarin red treatment for 10 min to quench background fluorescence. Sections were rinsed with PBS, non-specific binding blocked with a 3 % (w/v) BSA solution and then incubated overnight at 4 °C with primary antibodies rabbit anti-human laminin (1:30) (Abcam, United Kingdom), rabbit anti-human fibronectin (1:50) (Abcam, United Kingdom) and rabbit anti-human type I collagen (1:50) (Abcam, United Kingdom). After repeated washes in PBS, samples were incubated with Alexa Fluor 488 donkey anti-rabbit secondary antibody (1:500) (Molecular probes, USA) for 45 min at room temperature. Cells were washed in PBS and cell nuclei were counterstained with DAPI. Sections were analyzed with an AxioImager Z1m microscope (Zeiss, Germany), and images were acquired and processed with Zeiss Zen software version 2.6 (Zeiss, Germany).

### 2.9 Cell sheet thickness quantification

Immunohistochemistry images were processed using ZEN 3.8 software (Zeiss, Germany) using the length tool (Fig. S1). To do so, images of type I collagen, the most abundant ECM protein, were used. CS thickness was measured in 3 different locations across 3 randomly selected fields of samples from 4 different populations, with 34 measurements per condition.

### 2.10 Masson trichrome staining

CS sections were stained with Masson's Trichrome (MT) kit (Bio-Optica, Italy) according to the manufacturer's instructions. Briefly, sections were deparaffinized with xylene, rehydrated in graded ethanol series and stained with four different stains. Weigert's iron hematoxylin is used for nuclei, picric acid is used for erythrocytes, a mixture of acid dyes is used for cytoplasm, and aniline blue is used for connective tissue. Afterwards, sections were dehydrated and mounted with resinous mounting medium Entellan® (Merck, Germany). Histological sections were analyzed under a Leica DM750 microscope (Leica, Germany).

### 2.11 Fast green staining

CS sections were stained with fast green FCF (Sigma-Aldrich, USA), as previously described [21]. Briefly, sections were deparaffinized with xylene, rehydrated in graded ethanol series, and nuclei counterstained with DAPI at 1:1000 for 10 min at RT. For fast green FCF staining, samples were dehydrated using a series of methanol-in-water solutions (30 %, 50 %, 70 %, and 100 % methanol; 1 min each). Samples were then incubated in 50 μg/mL of Fast Green FCF (Sigma-Aldrich, USA) in methanol for 30 min at RT. Afterwards, the sections were rinsed in pure methanol to remove unbound staining and mounted in DEPEX (SERVA, Germany). Histological sections were analyzed under a Zeiss LSM980 AiryScan 2 (Zeiss, Germany) confocal microscope with ZEN 3.8 software (Zeiss, Germany).

### 2.12 Atomic force microscopy

CSs were immobilized on a Petri dish with the help of an acetate sheet on top, with a central hole of 6 mm. All measurements were carried out in PBS at 37 °C, which is compatible with cell survival. The nanomechanical properties of CSs were analyzed with a NanoWizard 3 (JPK Instruments, Germany), using a conical tip (half cone angle 15°) under force mapping mode. Before analysis, the probe was calibrated under PBS using the contact-based method. For each sample 8 × 8 maps were recorded using square acquisition frames of 10 × 10 μm^2^. At least three force maps were collected at different sample positions. The Young modulus of each analyzed sample position was calculated from the corresponding force curve by fitting the Hertz model using JPK SPM Data Processing software (JPK Instruments) and a Poisson ratio fixed at 0.5.

### 2.13 Statistical analysis and proteomics data processing

All data refers to 4 independent donors (*n* = 4). Statistical analyses were performed using GraphPad Prism 9 software (GraphPad Software Inc.). Proteomics data were processed using OmicsPlayground platform [22]. Log CPM values for all proteins were then used for UMAP, heatmap, functional annotation, and differentially expressed protein analysis. Heatmaps display the top 50 proteins with the highest standard deviation. Differentially expressed proteins were analyzed using notrend.limma with an FDR of 0.05. Protein annotation was performed using the COMPARTMENTS data set.

## 3 Results

### 3.1 Hypothermia maintains protein abundance pattern

**Fig. 1:**
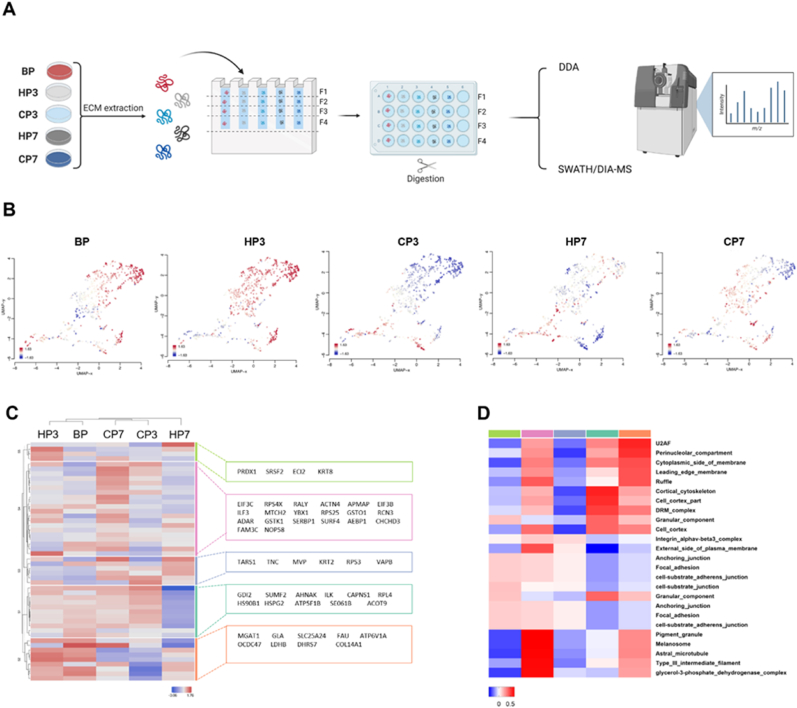
**Hypothermia maintains protein abundance pattern. A)** Schematic representation of CSs ECM extraction and subsequent proteomic analysis. **B)** UMAP plots for each condition colored by relative log-expression. Red indicates a protein is overexpressed in a specific group, blue that it is downregulated compared to the average values of all samples. Distance metric is covariance. **C)** Heatmap displaying unsupervised hierarchical clustering of top 50 proteins. Members of different clusters (indicated by bars on the right of the heatmap) are listed on the further right of the heatmap in colored areas. **D)** Heatmap of the functional annotation for each cluster were red indicates higher correlation and blue lower correlation. Annotation was performed using compartments data set.

To assess the effects of preservation on the ECM, proteomic analysis was carried out both before (BP) and after preservation (Fig. 1A). Firstly, the composition of the obtained proteome after decellularization was characterized. To identify features enriched in CSs proteome, differences in protein abundance were analyzed relative to the baseline (BP) for either hypothermic preservation (HP) or cryogenic preservation (CP), after 3 (HP3 or CP3) and 7 days (HP7 or CP7) (Fig. 1B). From the tested conditions, hypothermic preservation for 3 days maintained a protein abundance pattern closer to that observed before preservation, suggesting minimal alterations. However, prolonged preservation in hypothermic conditions led to a notable shift in the observed pattern. A significant shift in protein abundance was also observed for cryopreserved CSs, regardless of preservation time. Unsupervised hierarchical cluster analysis of the top 50 expressed proteins confirmed the similarity in protein abundance patterns between BP and HP3, manly given by proteins grouped in the orange cluster (Fig. 1C). To identify enriched features, proteins in the different clusters underwent annotation using a compartment data set (Fig. 1D). Enriched features indicate correlation with several cellular compartments, especially plasma membrane and adhesion molecules in all clusters. The orange cluster exhibits the highest correlations and highlights the similarities between BP and HP3, as well as the differences to the other groups.

### 3.2 Differentially expressed proteins

**Fig. 2:**
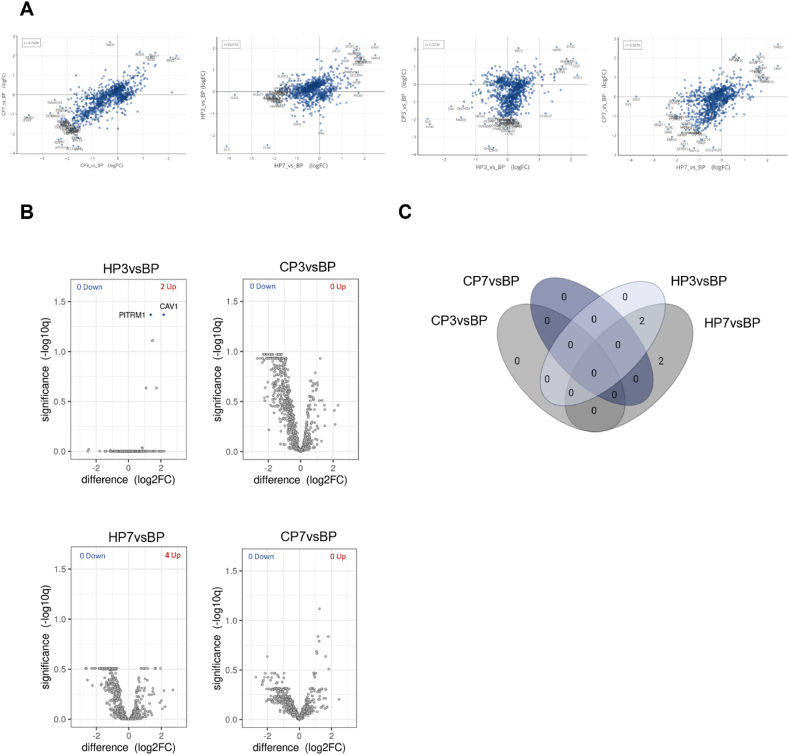
**Differentially expressed proteins. A)** Pairwise scatterplots of differential expression profiles for contrasts in analysis. Similar profiles show high correlation with points close to the diagonal. **B)** Volcano plot of differentially expressed proteins for each contrast (FDR = 0.05). **C)** Venn diagram with the distribution of the differently expressed proteins.

Protein abundance patterns were further characterized by correlation and differential expression analysis. Firstly, correlation analysis aimed at elucidating the relationships between preservation conditions and protein expression patterns (Fig. 2A). Specifically, when comparing CP3 and CP7, a strong positive correlation (r = 0.7429) was observed, suggesting a consistent pattern of protein abundance across cryogenic preservation conditions. HP7 and HP3 exhibited a moderate positive correlation (r = 0.4132), indicating some degree of similarity in protein abundance profiles between the two hypothermic preservation time periods. When comparing different preservation methods, a positive correlation was also observed, but its impact varied with increasing preservation times. Specifically, when comparing HP3 with CP3, a weak positive correlation was evident (r = 0.2739). In fact, it was the lowest among all comparisons. Interestingly, the correlation between different preservation methods increased with longer preservation times (r = 0.5276), suggesting a tendency for similar protein expression profiles with increasing preservation times regardless of the preservation methods. After examining the influence of preservation conditions on protein abundance patterns, an analysis focused on differential protein content was performed (Table S1). This analysis aimed to identify proteins that exhibit statistically significant differences in abundance between the experimental conditions investigated, using the levels before preservation - BP - as benchmark (Fig. 2B). The use of cryogenic preservation did not yield any significant differential abundances compared to the BP condition. Conversely, HP3 presented only two proteins with differential abundances compared to BP. This number increased to four after 7 days of hypothermic preservation. A Venn diagram was used to visually represent the overlap of differentially expressed proteins (Fig. 2C). Specifically, two proteins were observed to be differentially expressed in both HP3 and HP7 samples, with an additional two proteins differentially expressed in HP7. Among these proteins, only one was related with ECM, namely, EGF-containing fibulin-like extracellular matrix protein 2 (EFEMP2). EFEMP2 is part of the core matrisome, a subset of the matrisome, which is the collection of all proteins that constitute and interact with the ECM, according to the definition by Naba et al. [23]. This protein was then analyzed in more detail in the next section.

### 3.3 Matrisome protein abundance is preserved for all tested preservation methods

**Fig. 3:**
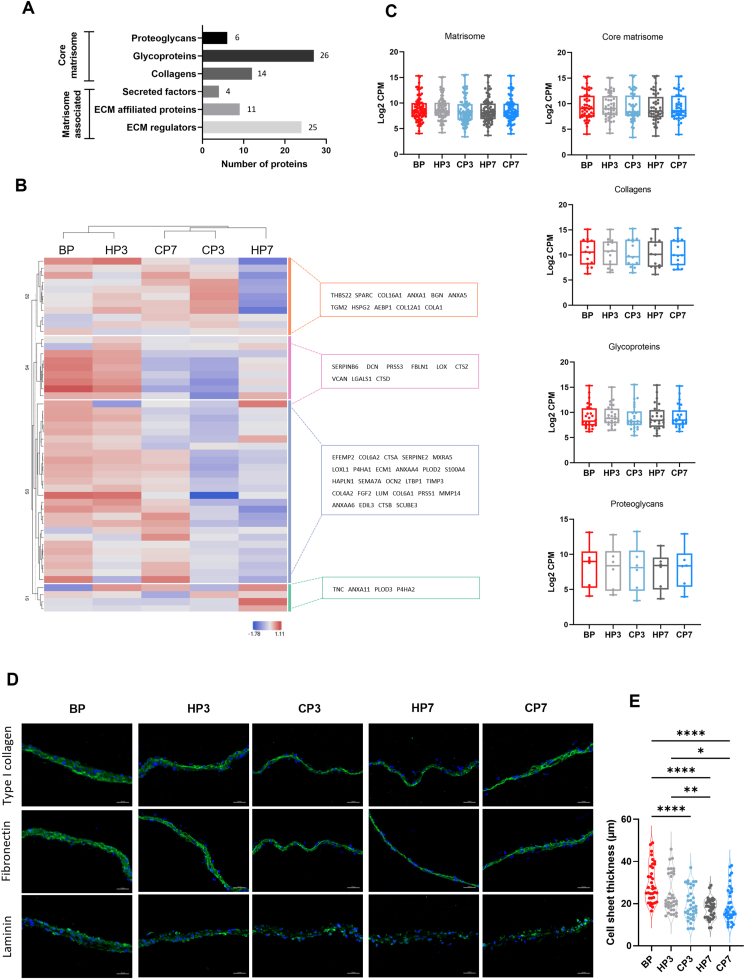
**Cell sheets matrisome profiling. A)** Total number of proteins, and number of identified matrisome proteins (left). Representation of each matrisome category, and respective number of ECM proteins identified (right). **B)** Heatmap displaying unsupervised hierarchical clustering of top 50 proteins of ECM. Members of different clusters (indicated by bars on the right of the heatmap) are listed on the further right of the heatmap in colored areas. **C)** Distribution plots of condition in the different categories of the matrisome as indicated. Results were analyzed using Kruskal-Wallis test with Dunn's multiple comparison post-test. **D)** Representative immunocytochemistry images of expression of major ECM proteins type I collagen, fibronectin and laminin (all in green). Cell nuclei was counterstained with DAPI (blue) Immunocytochemistry was performed after removing cultures from preservation. Scale bar: 50 μm. **E)** Quantification of CS thickness before and after preservation. Data are presented as violin plot illustrating the kernel density distribution frequency of CS thickness and analyzed using one-way ANOVA with Tukey multiple comparison post-test (∗p < 0.0332, ∗∗p < 0.0021 and ∗∗∗∗p < 0.0001).

To unveil potential effects of the preservation protocols on ECM-related proteins, the CSs matrisome was explored. Of 1034 identified proteins, 86 were recognized as ECM components based on the human matrisome dataset [24]. A total of 46 core matrisome proteins were identified, comprising 6 proteoglycans, 26 glycoproteins, and 14 collagens, along with 40 ECM-associated proteins, including 4 secreted factors, 11 ECM-affiliated proteins, and 25 ECM regulators (Fig. 3A) (Table S2). Among the detected collagens were various fibrillar types, such as types I, III, V, and XI, as well as non-fibrillar types, such as types IV, VI, VIII, XII, and XVI. Additionally, key constituents of the core matrisome, such as fibronectin and laminin, were identified within the glycoproteins classification. To investigate changes to the matrisome protein signature before and after preservation, an unsupervised hierarchical cluster analysis was performed on the top 50 proteins found in the ECM (Fig. 3B). The analysis revealed a closer matrisome protein pattern for baseline (BP) and HP3, while remaining conditions were clustered farther away, indicating more changes to matrisome protein abundance for these latter groups (Fig. 3B). Given the structural importance of core matrisome proteins, an initial analysis of their abundances for each condition revealed no statistically significant differences between the conditions (Fig. 3C). This was also the case when subdividing core matrisome proteins according to protein types. Immunohistochemistry analysis (Fig. 3D) corroborated these findings, showing no apparent changes in protein expression of key ECM constituents collagen type I, fibronectin, and laminin before and after preservation, regardless of the preservation method applied, which was further supported by dot blot analysis (Fig. S2). However, a reduction in CS thickness was observed post-preservation, suggesting direct damage to ECM structure due to the preservation processes rather than changes to protein abundances (Fig. 3E).

### 3.4 Cryopreservation alters matrix organization and structure

**Fig. 4:**
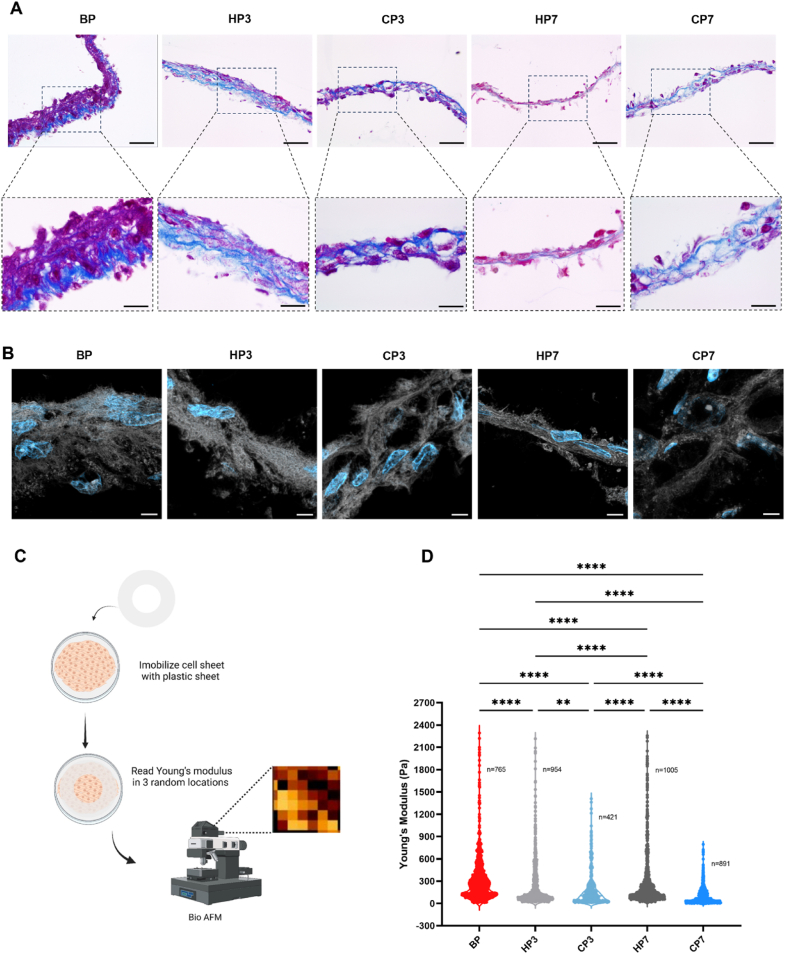
**Cryopreservation alters matrix organization and structure. A)** Representative Masson Trichrome staining images. Collagen is stained in blue and cells and other components in red. Staining was performed after removing cultures from preservation. Scale bar: 50 μm (top) and 20 μm (bottom) **B)** Representative fast green staining images. Collagen structures depicted in white and cell nuclei counterstained with DAPI (blue) Scale bar: 5  μm **C)** Schematic representation of Bio AFM methodology. **D)** ECM mechanical properties given by Bio AFM analysis. young's modulus (Pa) values were obtained before and after removing cultures from preservation. Data are presented as violin plot illustrating the kernel density distribution frequency of young's modulus and analyzed using one-way Kruskal–Wallis test with Dunn's multiple comparison post-test (∗∗p < 0.0021 and ∗∗∗∗p < 0.0001).

After observing changes in thickness following preservation, other structural and mechanical changes to CS were investigated. Histological analysis with MT staining revealed a pronounced disruption of ECM uniformity following cryogenic preservation, especially for prolonged preservation periods, aligning with the observed reduction in CS thickness (Fig. 4A). Some degree of ECM damage was also observed for the HP7 condition although not as severe as for the cryogenic conditions. As suggested by the other data, HP3 was the condition that better maintained ECM structure in comparison with the baseline BP condition. A finer analysis of collagen structure made using confocal microscopy after fast green staining, revealed fragmentation and disruption in collagen uniformity, corroborating the macroscopic structural alterations observed above (Fig. 4B). However, when examining structural integrity at the nanoscale, no differences were observed between conditions, suggesting that the primary structural damage following preservation occurs at a macroscopic rather than nanoscale level (Fig. S3). Notably, CSs preserved under hypothermic conditions maintained the organization of collagenous structures similar to the BP state, which was furthermore reflected in their mechanical properties measured by BioAFM (Fig. 4C). Young's modulus was assessed through nanoindentation, where a probe indents the sample surface to generate a force-displacement curve. Three force maps were collected from distinct regions on each cell sheet, with each map containing multiple measurements (indentations) across a defined area. Each point on the resulting graph represents a single measurement, illustrating stiffness distribution within the cell sheet. The observed variation in Young's modulus reflected the heterogeneous stiffness of cell sheet components: softer cellular regions yield lower modulus values, while stiffer, aligned collagen fibrils produce higher values. Across all preservation conditions, CS stiffness decreased compared to the BP condition. However, both the HP3 and HP7 conditions presented a kernel density distribution frequency similar to BP condition, indicating a better preservation of the stiffer components of CSs, in marked contrast with CSs preserved under cryogenic temperatures (Fig. 4D).

## 4 Discussion

The ECM plays a crucial role in CS technology, providing the structural framework necessary for cell attachment, proliferation, and differentiation [1]. As the primary component of CSs, the ECM dictates their mechanical properties and determines their ability to form functional tissue-like constructs. Given the delicate nature of CSs and their reliance on the ECM for structural support and signaling cues, appropriate preservation methods are essential to streamline their clinical implementation. Cryogenic preservation, considered the gold standard, and hypothermic preservation are two possible preservation approaches [16,25]. Yet, no direct comparison between these methods has been made for this purpose. Therefore, in this study, we aimed to comprehensively characterize the compositional, structural, and mechanical impact of cryogenic and hypothermic preservation on CSs ECM.

The chosen preservation timeframe was selected because it incorporates the periods during which maximal damage occurs for both preservation strategies. If, on the one hand, hypothermic preservation is inherently time-sensitive, as cellular metabolism is only partially suppressed and biochemical activity continues, on the other, it is widely known that in cryopreservation damage primarily occurs during the freeze-thaw process [26]. Furthermore, examining the ECM composition of CSs before and after preservation is essential for assessing the preservation's effects.and reducing sample complexity is vital to better visualize the proteins of interest [27,28]. The applied decellularization process removed a substantial portion of the cellular content and allowed the enrichment of the sample in ECM proteins. Of these, the matrisome refers to the collection of proteins that form or are associated with the ECM. This includes both the structural ECM proteins such as collagens, fibronectins, and elastins, known as the core matrisome, and the broader set of matrisome-associated proteins, comprising enzymes, growth factors, cytokines, and other molecules that interact with or regulate the ECM [23,29]. While the identified ECM proteins encompassed both core matrisome and matrisome-associated categories, this analysis primarily focused on core matrisome proteins, driven by the key role these structural proteins play in maintaining CS integrity, and thus their stability and functionality. Among these structural proteins, collagens are the most abundant, representing over 30 % of the ECM content [30]. The herein identified fibrillar collagens play a crucial role in defining the structural architecture and mechanical properties of CSs [31], while proteoglycans add strength and structure to the ECM. Among the observed glycoproteins, fibronectins and laminins are critical for ECM assembly and cellular adhesion by interacting with integrins, growth factors, cytokines, and other ECM molecules [32]. Our analysis also revealed smaller families of ECM proteins with specific roles in structural organization. This is the case of fibulin-2 and its involvement in the formation of large proteoglycan networks, and fibrillins, that provide tissue extensibility and linking to elastic fibers [33]. After identifying these key ECM proteins, the influence of preservation methods on their relative content was investigated. Looking at relative protein abundance using a differentially expressed protein analysis revealed no significant differences between conditions for ECM proteins, except for EFMP2, upregulated after 7 days under hypothermic temperatures. While EFEMP2 is described as interacting with other ECM components and cell surface proteins, it is not thought to play a preponderant role in ECM structure and, therefore, has not been further explored. When examining the expression patterns of the most abundant ECM proteins using immunohistochemistry, the results corroborated the findings from the differential expression analysis. With ECM protein content remaining relatively unchanged, alterations in ECM structure following preservation were analyzed. Unlike cryopreservation, hypothermic temperatures offer a milder alternative that avoids cellular and structural damage caused by ice nucleation and osmotic stress from cryoprotectants [34,35]. Although numerous hypothermic preservation solutions exist, most are tailored for whole organ preservation. Specifically developed for TE products, HTS is capable of maintaining ionic and osmotic balance, inhibiting acidosis, and preventing cell swelling [36,37]. Additionally, its scavenging capacity effectively reduces the production of reactive oxygen species (ROS), preventing the activation of various cellular stress pathways connected to oxidative stress, which in turn can lead to cell death [38]. These attributes collectively play a fundamental role in preserving cellular viability and ECM integrity under hypothermic conditions. This was confirmed in the present study, with CSs preserved with HTS better maintaining their structural integrity in comparison with cryopreserved CSs. However, a reduction in CSs thickness was noted for longer preservation times. While the precise factors driving this reduction are elusive, similar phenomena have already been described in the literature for other cold preservation scenarios [39,40]. Indeed, the results herein disclosed for the cryogenic preservation group showed a reduction in CS thickness similar to that observed for extended hypothermic preservation. Unlike intracellular ice, extracellular ice formation is typically considered benign for single-cell cryogenic preservation. However, its effects on densely packed cell-ECM structures can be detrimental. The intricate organization of ECM fibers and their interactions within the tissue architecture make them particularly susceptible to damage during cryopreservation [41]. As ice crystals grow in the extracellular space during cryopreservation, they alter the structure of ECM, especially affecting collagen and elastin organization [42]. This disruption to the ECM structure is significant, as it has been identified as the cause for the rapid deterioration observed in some implanted heart valves after cryopreservation [12,43,44]. Despite being less densely packed than most tissues, CSs exhibit similar structural features. Previous studies have revealed that cryopreservation can have notable effects on the original layered structure of CSs. Specifically, research on corneal epithelial CSs has documented evidence of ECM structural damage following cryopreservation [45]. Similarly, our results demonstrated ECM structural alterations after exposure to cryogenic temperatures. These changes in CS structure significantly impacted mechanical properties. which vary based on fiber orientation and the linkage and interaction of different fiber types [46]. Changes in alignment and interconnectedness of collagen fibrils with other ECM components significantly influence mechanical properties. Exposure to cryogenic temperatures decreased CS stiffness, which correlated well with the extensive damage to the collagen network shown by the fragmentation and discontinuity of ECM and collagen fibrils observed after MT staining and specific collagen staining. Nevertheless, although having performed measurements at various locations on the CS, it is essential to acknowledge that indentation techniques may not fully represent the dynamics of the entire tissue. Finally, the observed effects may vary depending on both the cell type and the number of CS layers. The nature of CS constructs depends on factors like cell proliferation rates and the quantity and composition of ECM produced by the cells, and thus sheets derived from different cell types, and especially those organized in multilayered structures, may respond differently to preservation methods. Multilayered constructs, in particular, represent more densely packed cell-ECM arrangements where the interactions among ECM fibers are more intricate, rendering them, on the one hand, potentially more susceptible to cryopreservation-induced damage, but, on the other, more robust and therefore potentially more resilient to such insult. This is something that should be specifically tested in future works, although we believe the overall conclusions of the present work are valid for CS constructs with low complexity and thickness.

## 5 Conclusions

This study provides comprehensive insights into the effects of cryogenic and hypothermic preservation on CS ECM composition as well as on its structure and mechanical properties. Our findings demonstrate that exposure to cryogenic temperatures resulted in significant alterations in ECM structure and, in particular, disruptions to collagen organization. Conversely, hypothermic preservation had a less pronounced effect on ECM structure, with CSs preserving, to a large extent, their original structural integrity and mechanical properties. The observed alterations in ECM structure have important implications for the clinical implementation of CS-based therapies. While no significant alteration in terms of ECM protein abundance was found, the notable ECM structural changes following cryogenic preservation can potentially impact CSs physiologic function, thereby affecting their efficacy CS when used clinically as therapies. This suggests that hypothermia may be the best alternative for preserving CS integrity and functionality from the fabrication site to the bedside.

## CRediT authorship contribution statement

**Sara Freitas-Ribeiro:** Writing – review & editing, Writing – original draft, Methodology, Investigation, Formal analysis, Data curation, Conceptualization. **Andreia F. Carvalho:** Investigation, Formal analysis. **Daniel B. Rodrigues:** Investigation, Formal analysis. **Luís Martins:** Formal analysis. **Ricardo A. Pires:** Investigation, Formal analysis. **Vera M. Mendes:** Validation, Investigation, Formal analysis. **Bruno Manadas:** Validation, Formal analysis. **Mariana Jarnalo:** Resources. **Ricardo Horta:** Resources. **Rui L. Reis:** Supervision, Resources. **Rogério P. Pirraco:** Writing – review & editing, Writing – original draft, Validation, Supervision, Methodology, Funding acquisition, Data curation, Conceptualization.

## Ethics approval and consent to participate

Human subcutaneous adipose tissue was obtained from surgical procedures performed at Centro Hospitalar Universitário São João, after patient's written informed consent, and in the scope of a collaboration protocol approved by the ethical committees of both institutions for this work (Comissão de Ética do Centro Hospitalar Universitário São João/University of Minho: 217/19; CEICVS 008/2019).

## Declaration of competing interest

Rui L. Reis is an associate editor for Bioactive Materials and was not involved in the editorial review or the decision to publish this article. The remaining authors declare that there are no competing interests.
