# Supplementary material for: Cryogenic, but not hypothermic, preservation disrupts the extracellular matrix of cell sheets
Source: Bioact Mater. 2024 Dec 25;46:301–10. doi: 10.1016/j.bioactmat.2024.12.019 (PMC11732602; doi:10.1016/j.bioactmat.2024.12.019)
Supplement: Multimedia component 1 [file mmc1.docx]

**Supplementary materials**

**Fig. S1.** Schematic representation of the measurements obtained from representative immunohistochemistry images using ZEN 3.8 software. Type I collagen immunohistochemistry was used. After selecting the length tool, cell sheets were measured in 3 different locations across 3 randomly selected fields of 4 different populations, with a total of 34 measures per condition.


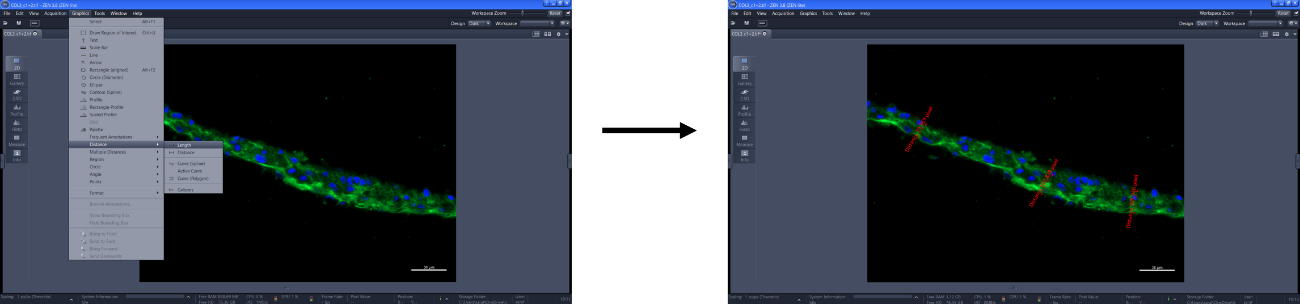


**Fig. S2. Dot blot analysis of major ECM proteins.** Dot blot analysis was used to semi-quantitatively assess the expression of major ECM proteins. For each condition, 2 µL containing 0.5 µg of protein were pipetted in duplicate onto a nitrocellulose membrane and allowed to dry overnight. After blocking with 5% (w/v) BSA in TBS, membranes were incubated overnight with primary antibodies rabbit anti-human laminin (1:700), rabbit anti-human fibronectin (1:2000), and rabbit anti-human type I collagen (1:2000) (all from Abcam, United Kingdom). Following a series of washes, membranes were incubated for 1 hour at room temperature with an IRDye 800CW anti-rabbit IgG secondary antibody (1:10,000) (LI-COR Biosciences, USA). Results were visualized using an Odyssey Fc Imaging System.


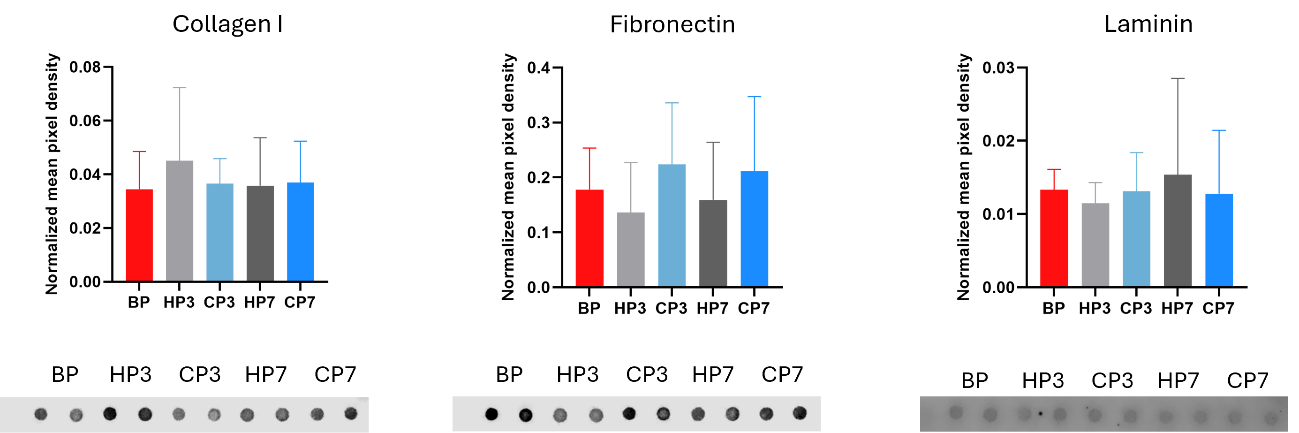


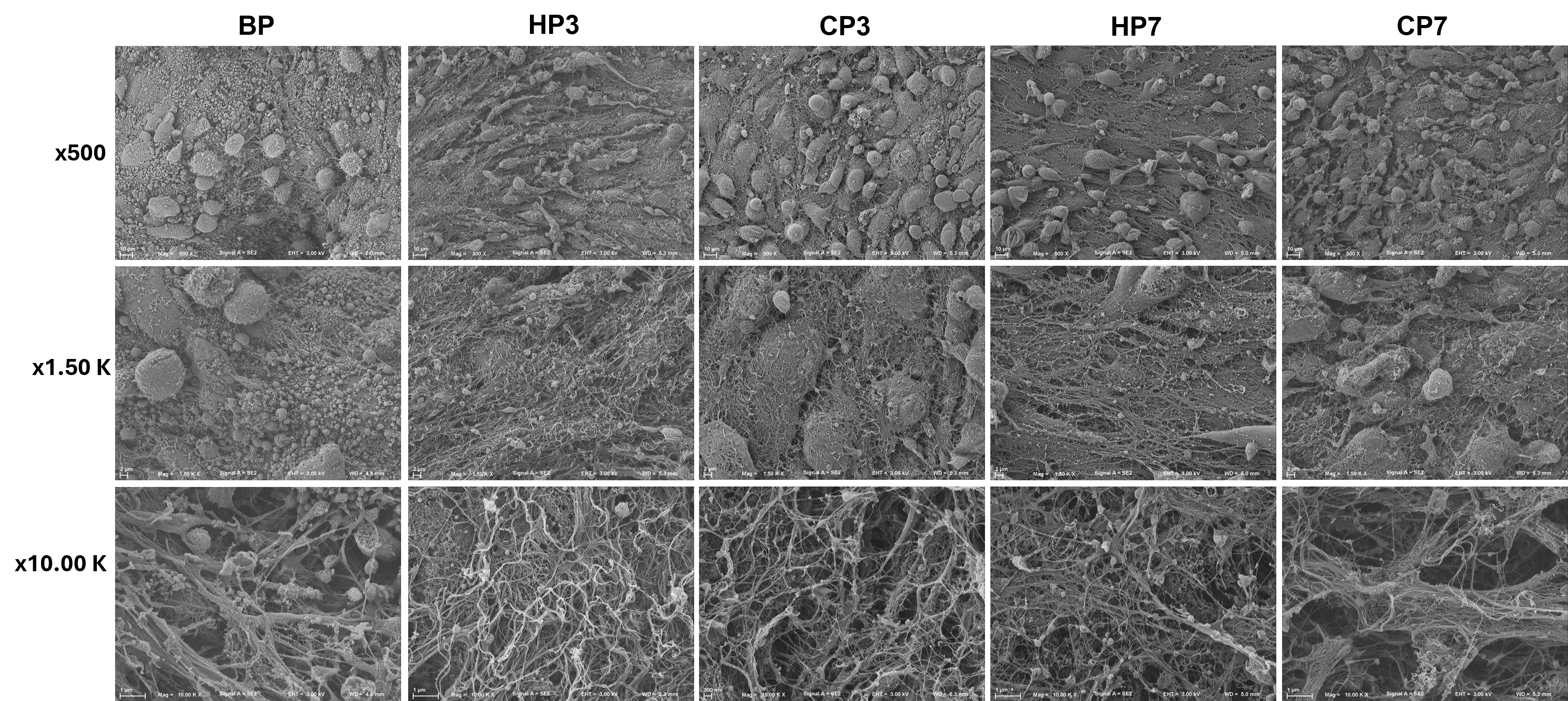
**Fig. S3. Scanning electron microscopy (SEM) images of cell sheets before and after preservation.** For ultrastructural analysis, cell sheets were fixed with a 2% buffered glutaraldehyde solution, and subsequently dehydrated using increasing concentrations of ethanol (20%, 40%, 60%, 80%, 90%, 95% and 100%). Cell sheets were then dried using an autosamdri-815 critical point dryer (Tousimis, USA). Images were acquired with different magnifications using a high-resolution field emission SEM (AURIGA Compact, Zeiss, Germany), operating at an accelerating voltage of 3 kV.

**Table S1.** Differentially expressed proteins across different comparisons**.**

| **Differentially expressed proteins** | | | | | | | |
| --- | --- | --- | --- | --- | --- | --- | --- |
| HP3vsBP | | CP3vsBP | | HP7vsBP | | CP7vsBP | |
| UP | Down | UP | Down | UP | Down | UP | Down |
| CAV1 |  |  |  | CAV1 |  |  |  |
| PITRM1 |  |  |  | PITRM1 |  |  |  |
|  |  |  |  | TOMM70 |  |  |  |
|  |  |  |  | EFEMP2 |  |  |  |

**Table S2.** Identified matrisome proteins

| **Core matrisome** | | | **Matrisome associated** | | |
| --- | --- | --- | --- | --- | --- |
| Collagens | Glycoproteins | Proteoglycans | ECM affiliated proteins | ECM regulators | Secreted factors |
| COL11A1 | AEBP1 | BGN | ANXA1 | CTSA | FGF2 |
| COL12A1 | DPT | DCN | ANXA11 | CTSB | S100A4 |
| COL14A1 | ECM1 | HAPLN1 | ANXA4 | CTSD | S100A11 |
| COL16A1 | EDIL3 | HSPG2 | ANXA5 | CTSZ | SCUBE3 |
| COL1A1 | EFEMP2 | LUM | ANXA6 | ITIH2 |  |
| COL1A2 | EMILIN1 | VCAN | LGALS1 | LOX |  |
| COL3A1 | FBLN1 |  | LGALS3 | LOXL1 |  |
| COL4A2 | FBLN2 |  | LMAN1 | LOXL2 |  |
| COL5A1 | FBN1 |  | SEMA7A | MMP14 |  |
| COL5A2 | FN1 |  | LMAN2 | P3H1 |  |
| COL6A1 | HMCN1 |  | ANXA2 | P3H3 |  |
| COL6A2 | LAMC1 |  |  | P4HA1 |  |
| COL6A3 | LTBP1 |  |  | P4HA2 |  |
| COL8A1 | LTBP2 |  |  | PLOD1 |  |
|  | MFGE8 |  |  | PLOD2 |  |
|  | MXRA5 |  |  | PLOD3 |  |
|  | NID2 |  |  | PRSS1 |  |
|  | POSTN |  |  | PRSS3 |  |
|  | PXDN |  |  | SERPINB6 |  |
|  | SPARC |  |  | SERPINE2 |  |
|  | TGFBI |  |  | SERPINH1 |  |
|  | THBS1 |  |  | TGM2 |  |
|  | THBS2 |  |  | TIMP3 |  |
|  | TINAGL1 |  |  | P3H4 |  |
|  | TNC |  |  | P4HB |  |
|  | TNXB |  |  |  |  |
